# Construction of Novel Polymerizable Ionic Liquid Microemulsions and the In Situ Synthesis of Poly(Ionic Liquid) Adsorbents

**DOI:** 10.3390/nano9030454

**Published:** 2019-03-18

**Authors:** Aili Wang, Shuhui Li, Hou Chen, Ying Liu, Xiong Peng

**Affiliations:** 1 School of Chemistry and Material Science, Ludong University, Yantai 264025, China; lsh5354@163.com (S.L.); 15563833132@163.com (Y.L.); 2 Guangdong Provincial Key Lab of Green Chemical Product Technology, Guangzhou 510640, China; cepeng.xiong@mail.scut.edu.cn

**Keywords:** polymerizable ionic liquid microemulsions, poly(ionic liquid)s, adsorption

## Abstract

This paper reports the successful construction of novel polymerizable ionic liquid microemulsions and the in situ synthesis of poly(ionic liquid) adsorbents for the removal of Zn^2+^ from aqueous solution. Dynamic light-scattering data were used to confirm the polymerization media and to illustrate the effect of the crosslinker dosage on the droplet size of the microemulsion. FTIR and thermal analysis were employed to confirm the successful preparation of the designed polymers and characterize their thermostability and glass transition-temperature value. The optimization of the adsorption process indicates that the initial concentration of Zn^2+^, pH, adsorbent dosage and contact time affected the adsorption performance of poly(ionic liquid)s toward Zn^2+^. Furthermore, our research revealed that the adsorption process can be effectively described by the pseudo second-order kinetic model and the Freundlich isotherm model.

## 1. Introduction

Poly(ionic liquid)s (PILs), which possess ionic liquid species in each of the repeating units, have attracted much attention in the field of polyelectrolyte preparation, porous membrane synthesis, adsorption and separation [1,2]. Among these applications, the adsorption of organic pollutants and metal ions in industrial wastewater has received increased attention from researchers [3]. PILs are polymerized from ionic liquid monomers, and thus possess the excellent physical and chemical properties of ionic liquids, such as molecular designability, negligible evaporation, and excellent thermal stability [1,4,5,6]. Moreover, PILs have overcome most of the disadvantages of ionic liquids, such as the high viscosity, recycling difficulty, and consequent toxic and corrosive risk [3,7].

Earlier research on PILs focused on their adsorption performance towards organics with ionic groups or aromatic nucleus, such as anionic dyes and triazole fungicides [8,9]. Currently, PILs are also showing an increased potential for heavy metal ion adsorption [10]. Interestingly, the “designability” of the ionic liquid monomers makes hydrophobic anions inducible into the molecular chain of PILs to introduce hydrophobic polymers, which increases their reusability for adsorbing heavy metal ions. Moreover, the adsorptive selectivity could be enhanced due to the “designability” of the monomers. The reported application of PILs focused on the removal of Cr(VI), but the adsorption capacity was unsatisfied. The hydrophobic PILs synthesized by Kong only showed a maximum adsorption capacity of 17.9 mg/g for hexavalent chromium [10]. The sulfonic acid-functionalized PILs synthesized by Khiratkar showed a higher adsorption capacity for hexavalent chromium in water, but there was still a gap compared to other adsorbents [11].

The traditional synthesis method of PILs was almost bulk-free radical polymerization at normal pressure. Our previous research showed that functional materials could be synthesized in situ within ionic liquid microemulsions [12]. Nowadays, microemulsions involving ionic liquids have attracted much attention in the world. As a “green solvent”, ionic liquid has been applied in various areas depending on its low volatility, designability, negligible vapor pressure and excellent stability advantages [13]. Previous research indicated that ionic liquids could replace any of the constituents in traditional microemulsions and form a new system named ionic liquid microemulsion [1,3,14,15]. This innovative finding not only enlarged the research scale of ionic liquid and microemulsion to a greater extent, but also promoted the development of the relevant subjects. Due to the controllability of the nanosized structure of the ionic liquid microemulsion droplets, nanomaterials such as TiO_2_, phosphate nanocrystals and polyaniline could be easily synthesized by controlling the microstructure of the designed microemulsions [16,17,18]. Moreover, ionic liquid monomers could also be used as the polar and/or non-polar phase of microemulsions, and these types of polymeric ionic liquid microemulsions could be used as an in situ synthesis media for PILs [19].

In the present study, we constructed a new type of ionic liquid microemulsion, in which all components were polymerizable. The polar phase was a mixture of 1-allyl-3-methylimidazolium chloride ([AMIM][Cl]) and cross-linking agent poly(ethylene glycol) diacrylate (PEGDA). The nonpolar phase was 1-allyl-3-methylimidazolium hexafluorophosphate ([AMIM][PF_6_]). Sodium 3-(allyloxy)-2-hydroxypropane-1-sulfonate (HAPS) was used as the surfactant, and no co-surfactant was involved. Compressed bulk free-radical polymerization was adopted to synthesize PILs in the polymerizable ionic liquid microemulsions (PILMs). The resulting PILs were used as an adsorbent for metal ions in aqueous solution. Zn(II), which mainly comes from metallurgical processing, electroplating and batteries industries [20], was selected here as the object metal ion. The effect of PEGDA dosage on the droplet size and size distribution of PILMs was investigated by dynamic light scattering (DLS). The influence of thermosynthesis time, synthesis temperature, adsorbent dosage, pH, initial concentration and contact time on the characteristic and adsorption capability of PILs for Zn(II) removal were investigated in detail.

## 2. Materials and Methods

### 2.1. Materials

[AMIM][PF_6_] (>99 wt%) and [AMIM][Cl] (>99 wt%) were purchased from Lanzhou Institute of Chemical Physics. HAPS (>99 wt%) was obtained from Hanerchem Ltd. PEGDA (Mn = 575), azobisisobutyronitrile (AIBN, >99 wt%) and zinc chloride (ZnCl_2_, >98 wt%) were purchased from Sigma Aldrich. Prior to the experiment, HAPS was vacuum-dried at 70 °C for 6 h to remove excess water. The other chemicals were used without further purification.

### 2.2. Methods

**Scheme 1:**
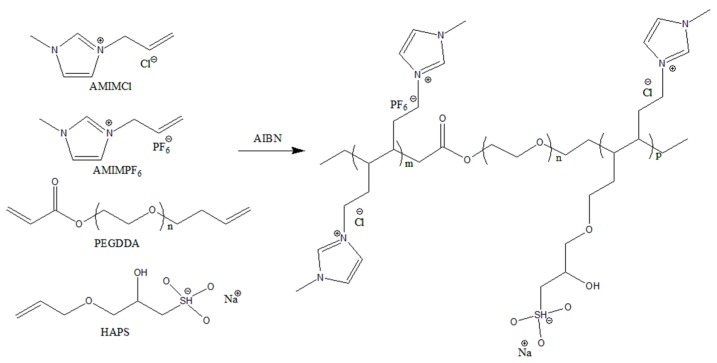
The synthesis process of PILs.

In a typical experiment, the polymerization process of PILs included the following steps: (1) [AMIM][PF_6_], [AMIM][Cl], HAPS and PEGDA at different mass ratios were mixed at 60 °C for 40 min under moderate magnetic stirring, and the microemulsions named PILM-1 to PILM-4 were obtained. (2) The mixture was transferred into a sealed Teflon-lined autoclave and subsequently kept at 80–120 °C for 12–28 h. (3) The reaction set was cooled to room temperature, and the obtained products were precipitated by ethyl acetate followed by deionized water three times. (4) The brown precipitate was collected by rotary evaporation and vacuum-dried overnight at 40 °C, and the final products, named PIL-1 to PIL-5, were obtained. The general recipe used in the synthesis of PILs is shown in Table 1. In addition, the synthesis process of the PILs is shown in Scheme 1.

### 2.3. Characterization of PILs

A Malvern Nano ZS was applied to determine the droplet size, size distribution and zeta potential of PILMs. A Perkin Elmer Spectrum 2000 was applied to determine the FTIR spectrum of PILMs. The thermogravimetric data of the PILMs were collected by a Netzsch STA449C F3 instrument at a heating rate of 10 °C/min under argon atmosphere from room temperature to 600 °C. Differential scanning calorimeter (DSC) analysis was performed on a Netzsch 204F1 calorimeter and carried out over a temperature range of −50 °C to 150 °C with a heating rate of 10 °C/min. The yield was obtained using the gravimetric method. The equilibrium degree of swelling (*E_s_*) of the PILs was measured by gravimetry.

*E_s_* is defined as:

$$E_{s}=\frac{W_{s}}{W_{d}},$$

where *W_s_* is the weight of the PILs after equilibrium swelling, and *W_d_* is the dry weight of the PILs.

In order to evaluate the PILs’ adsorption capacities toward Zn(II) in aqueous solution, the batch adsorption experiments were performed as follows. In a typical experiment, the mixtures of the designed PILs and 20 mL of 50 mg/L Zn(II) solution were added into a series of 100 mL Erlenmeyer flask with a glass stopper. At a constant temperature of 25 ± 1 °C in a thermostatic water bath, the samples were shaken at about 100 rpm for certain contact time. The Zn(II) ion concentrations of the after-adsorbed samples were determined by flame atomic absorption spectroscopy (AAS). The adsorption capacities ($q_{e}$ in mg/g) of the designed PILs were calculated according to Equation (1):

(1) $$q_{e}=\left(C_{0}-C_{e}\right)\times \frac{V}{m},$$

where $C_{0}$ is the initial concentrations of Zn(II) ion (mg/L), $C_{e}$ is the equilibrium concentrations of Zn(II) ion (mg/L), V is the volume of Zn(II) solution (L), and m is the dosage of PILs (g). 

## 3. Results and Discussion

### 3.1. Droplet Size and Size Distribution

**Figure 1:**
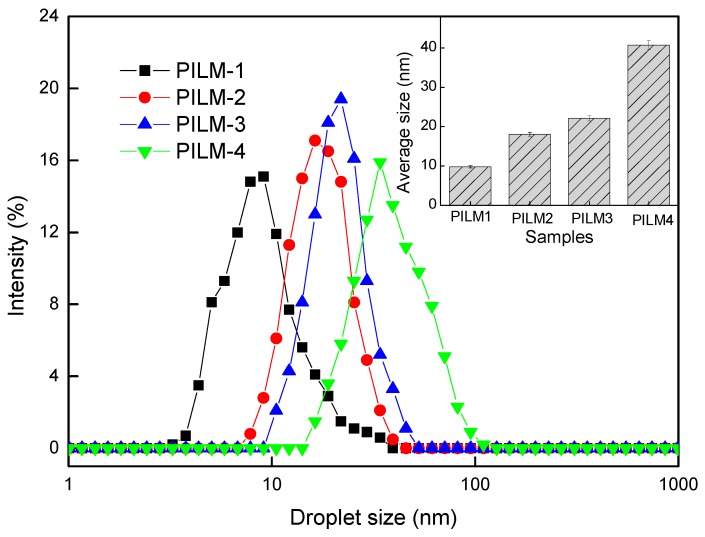
Size and size distribution of the [AMIM][Cl]/[AMIM][PF_6_]/PEGDA/HAPS microemulsion with different amounts of PEGDA.

Transparent PILMs involving [AMIM][Cl], [AMIM][PF_6_], PEGDA and HAPS were constructed successfully. Normally, dynamic light scattering (DLS) is used to determine the droplet size and size distribution of microemulsions containing ionic liquids [21]. Herein, the average droplet size and size distribution of the [AMIM][Cl]/[AMIM][PF_6_]/PEGDA/HAPS microemulsions with different amounts of PEGDA were collected and shown in Figure 1. It can be noticed that the size distribution of each PILMs was monodispersed, and the average size ranged from 10–40 nm, which was in accordance with the microemulsion’s nature (<150 nm) [22]. Besides, with the increasing amount of PEGDA, the average size of PILMs increased slightly, and this could be attributed to the droplet structure of PILMs. According to the molar weight of each component in the construction of the PILMs, PEGDA was stable as the inner phase of the microemulsion when the ionic liquids acted as the outer phase. Therefore, the increment dosage of PEGDA enlarged the size of the inner core, and hence, increased the droplet size. The zeta potential values of the [AMIM][Cl]/[AMIM][PF_6_]/PEGDA/HAPS microemulsions were −24.13 ± 0.85 mV indicating that the polymerizable ionic liquid microemulsions had good stability.

### 3.2. FTIR Analysis

**Figure 2:**
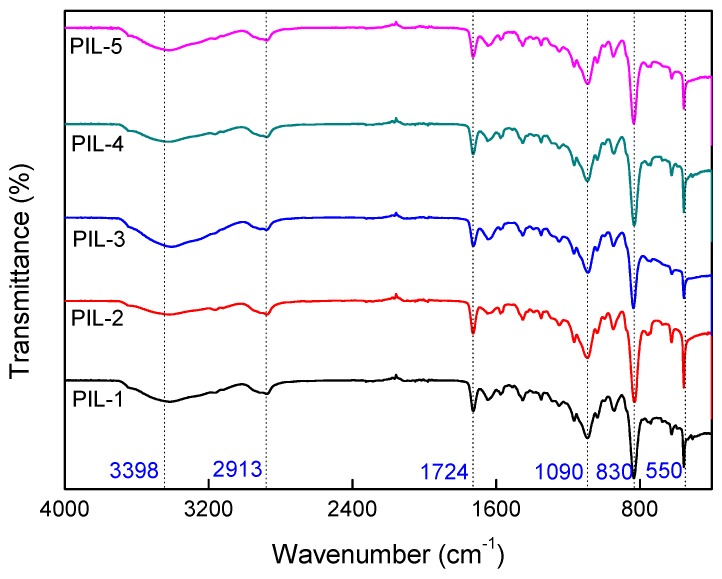
FTIR spectra of PILs synthesized under different temperature.

The in situ synthesized PILs with different reaction temperatures—80 °C, 90 °C, 100 °C, 110 °C and 120 °C, represented by PIL-1, PIL-2, PIL-3, PIL-4 and PIL-5, respectively—were characterized by Fourier transform infrared spectroscopy (FTIR). The results are shown in Figure 2, where the adsorption peak around 1724 cm^−1^ and 1090 cm^−1^ can be attributed to the stretching vibration of C=O and C–O in PEGDA, respectively. The peak that appeared at 2913 cm^−1^ was attributed to the C–H stretching vibration in the saturated carbon chain. The absorption peak located at 830 cm^−1^ and 550 cm^−1^ was characteristic of PF_6_^−^ and Cl^−^, respectively, which suggested the successful synthesis of the PILs within the PILMs.

### 3.3. Thermal Analysis of PILs

**Figure 3:**
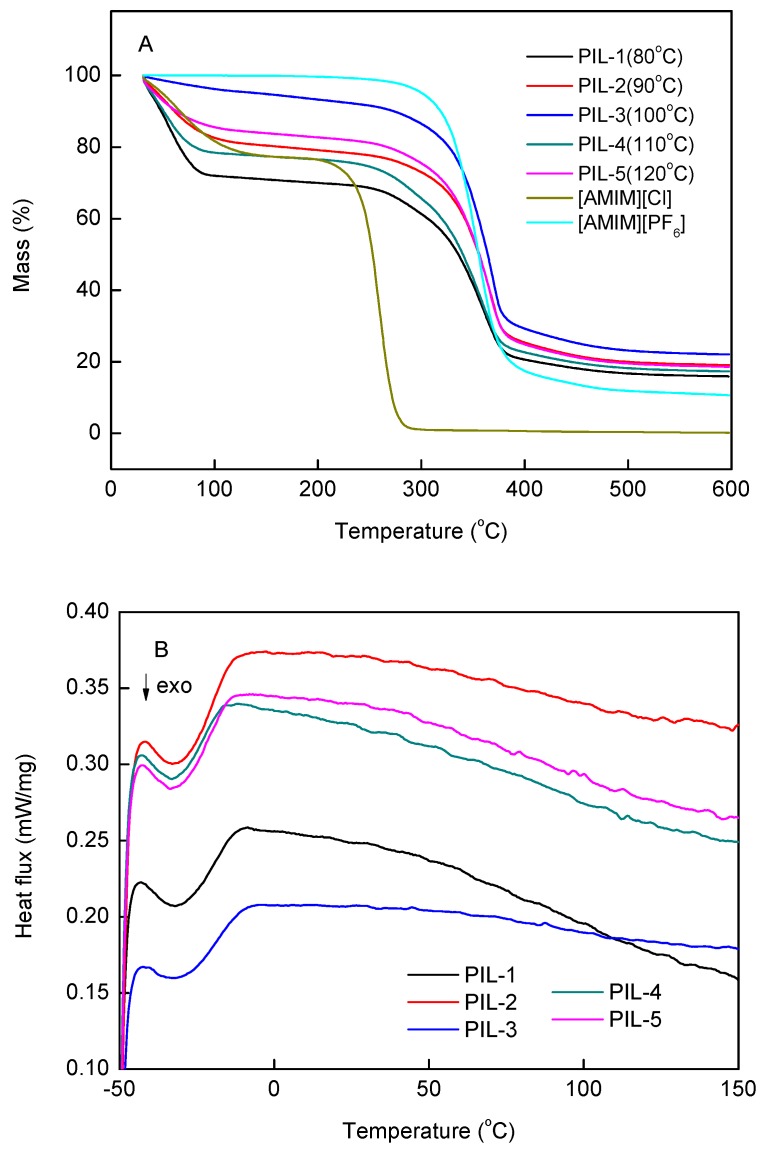
Thermogravimetric curves of [AMIM][Cl], [AMIM][PF_6_] and PILs. (**A**) DSC curves of PILs (**B**).

The thermogravimetry curves of [AMIM][Cl], [AMIM][PF_6_] and the as-prepared PILs are presented in Figure 3A. Each PIL weight loss exhibited two distinct temperature scopes. Due to the evaporation of the absorbed water, the PILs showed an initial stage of weight loss in the range of room temperature to 100 °C. This absorbed water was mainly combined with chlorine salt because of the water-absorbing quality of [AMIM][Cl]. The second stage of weight loss was found in the range of 300 °C to 400 °C, which can be ascribed to the fracture of the imidazole ring in the PILs as well as the [AMIM][Cl] and [AMIM][PF_6_] monomers. These results showed that PILs synthesized within the PILMs had combined the thermogravimetry characteristics of [AMIM][Cl] and [AMIM][PF_6_]. The water resistance of PILs synthesized over 80 °C manifested improvements to a certain extent compared to [AMIM][Cl]. PIL-3, which was synthesized at 100 °C, showed the best thermogravimetry performance within the range of study.

The swelling behavior of PILs in Zn(II) solutions was further investigated with the gravimetry method. As shown in Table 2, the designed PILs could be well-swelled in solutions, and the complexation of the ester group of PEGDA with Zn(II), and the hydroxy and the sulfonic group of HAPS with Zn(II) occurred simultaneously. Since five PILs had the same ionic liquid and crosslinker content, the effect of the synthesis temperature of the PILs on *E_s_* was not obvious. 

Figure 3B shows the DSC curves of different PILs. The results showed that the glass transition temperatures (*T_g_*) of the PILs were approximately −30 °C, which indicated their phase transformation point. Altering the reaction temperature of the PILs did not produce obvious changes in the *T_g_* position. 

### 3.4. Influence on the Adsorption Potential of PILs

**Figure 4:**
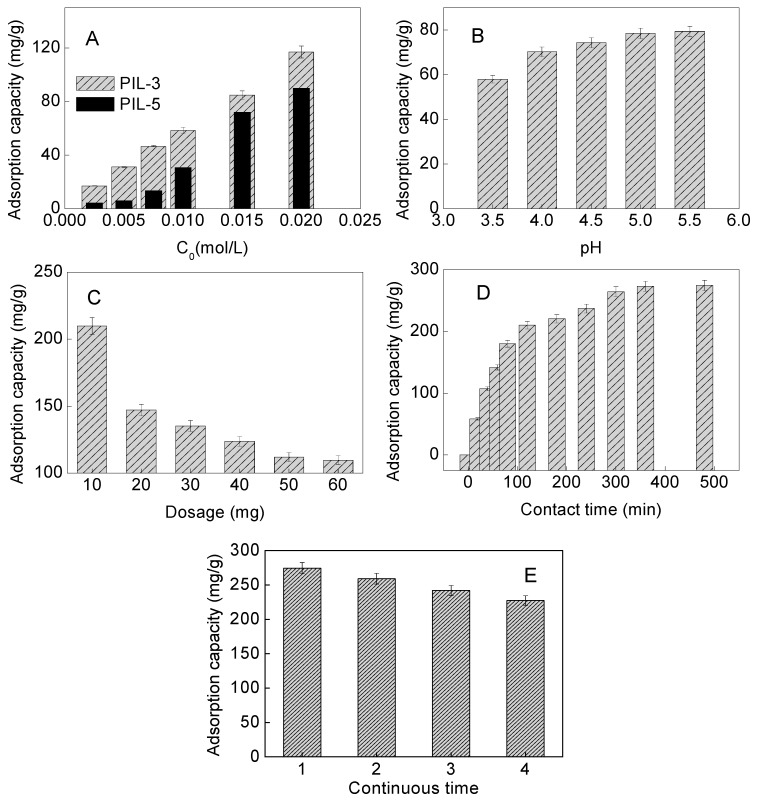
The effect of C_0_ on the adsorption performance of PIL-3 and PIL-5 for Zn^2+^ (**A**), the effect of pH (**B**), adsorbent dosage (**C**), contact time (**D**) and continuous times (**E**) on the adsorption performance of PIL-3 for Zn^2+^.

The as-prepared PILs were employed in the removal of Zn^2+^ from water, and PIL-3 was selected as the model adsorbent because of its distinct properties mentioned above. In addition, the adsorption performance of PIL-5 was investigated for comparison (volume—25 mL, pH—4.5, dose—10 mg, time—8 h and temperature—25 ± 1 °C). As shown in Figure 4A, within the studied range of the initial concentration of Zn^2+^ (C_0_), the PIL-3 sample exhibited better adsorption performance than PIL-5. Moreover, with the increment of C_0_, the adsorption capacities of both PIL samples toward Zn^2+^ increased gradually. 

The effects of pH and adsorbent dosage on the adsorption of Zn^2+^ onto PIL-3 were investigated, and the results are provided in Figure 4B,C, respectively. It can be observed that the saturated adsorption capacity grew up slightly with the increase in solution pH from 3.5 to 5.5, suggesting that electrostatic attraction played a role in the adsorption process (C_0_—0.01 M, volume—25 mL, dose—10 mg, time—8 h and temperature—25 ± 1 °C). Notably, lowering the adsorbent dosage brings about the increasing of adsorption capacity, which can be ascribed to the excess adsorption sites (C_0_—0.01 M, volume—25 mL, pH—4.5, time—8 h and temperature—25 ± 1 °C). 

Figure 4D shows the adsorption behavior of PIL-3 with Zn^2+^ as a function of contact time (C_0_—0.01 M, volume—25 mL, pH—4.5, dose—10 mg and temperature—25 ± 1 °C). The adsorption capacity of PIL-3 toward Zn^2+^ increased rapidly from 58.34 to 179.81 mg/g when the contact time was raised from 20 to 80 min. On further raising of the contact time to 300 min, the adsorption capacity increased slowly. In order to achieve sufficient adsorption, 480 min was selected as the optimum contact time. The influence on the adsorption potential of the designed PILs indicated that the maximum adsorption capacity of the PILs for Zn(II) was greater than the values of various reported adsorbents [23,24,25,26,27,28].

In addition, PIL-3 was easily released in 0.1 mol/L HCl after absorbing Zn^2+^ from the water. After washing with deionized water and drying in a vacuum at 50 °C, PIL-3 was able to be continuously used more than three times without a significant decrease in Zn^2+^ removal efficiency (<12%), as shown in Figure 4E.

### 3.5. Adsorption Kinetics

**Figure 5:**
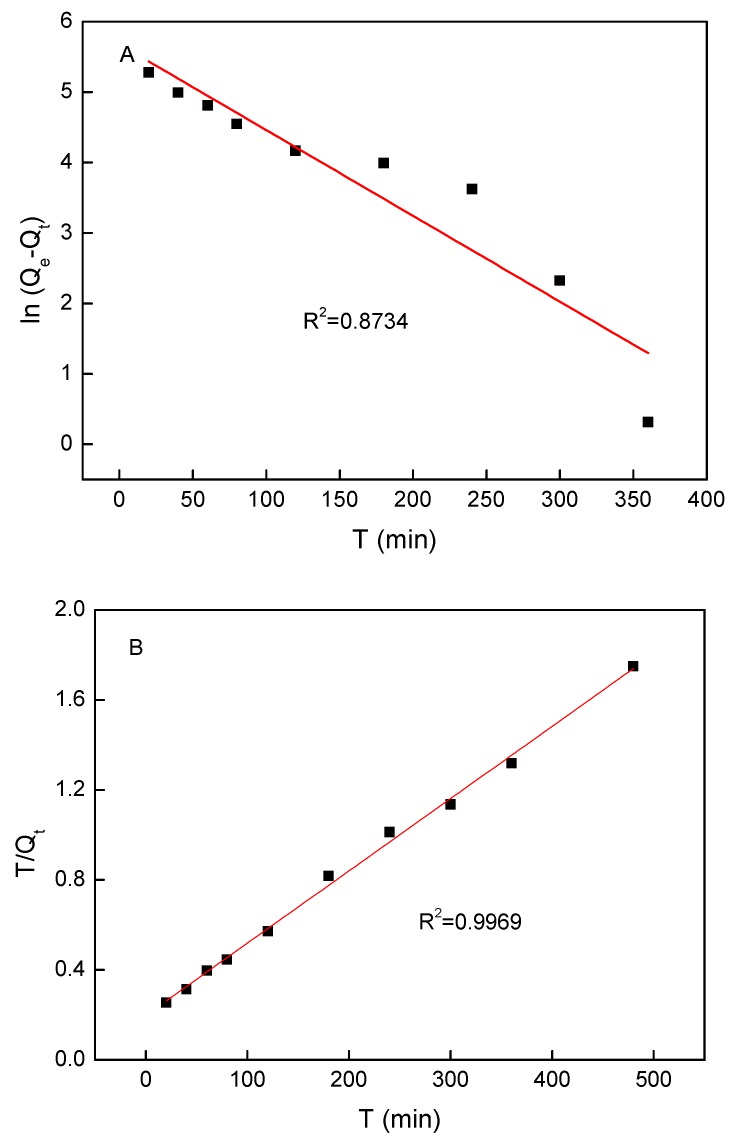
Pseudo-first-order kinetic model fitting (**A**) and pseudo-second-order kinetic model fitting (**B**) of the adsorption process for PIL-3 toward Zn^2+^.

The sorption kinetics for the adsorption of Zn^2+^ onto PIL-3 was investigated, and the resulting data were fitted in the pseudo-first-order and pseudo-second-order kinetic models, as shown in Figure 5. The calculated kinetic parameters are shown in Table 3. Obviously, the adsorption process was more aligned with the pseudo-second-order model (R^2^ = 0.997) than with the pseudo-first-order model (R^2^ = 0.874). Besides, for the pseudo-second-order model, the calculated saturated adsorption capacity value was also closer to the experimental data. These findings indicate that the ion exchange and physical adsorption on the surface and the hydroxyl complexation may control the valence forces between PIL-3 and Zn^2+^.

### 3.6. Adsorption Isotherms

**Figure 6:**
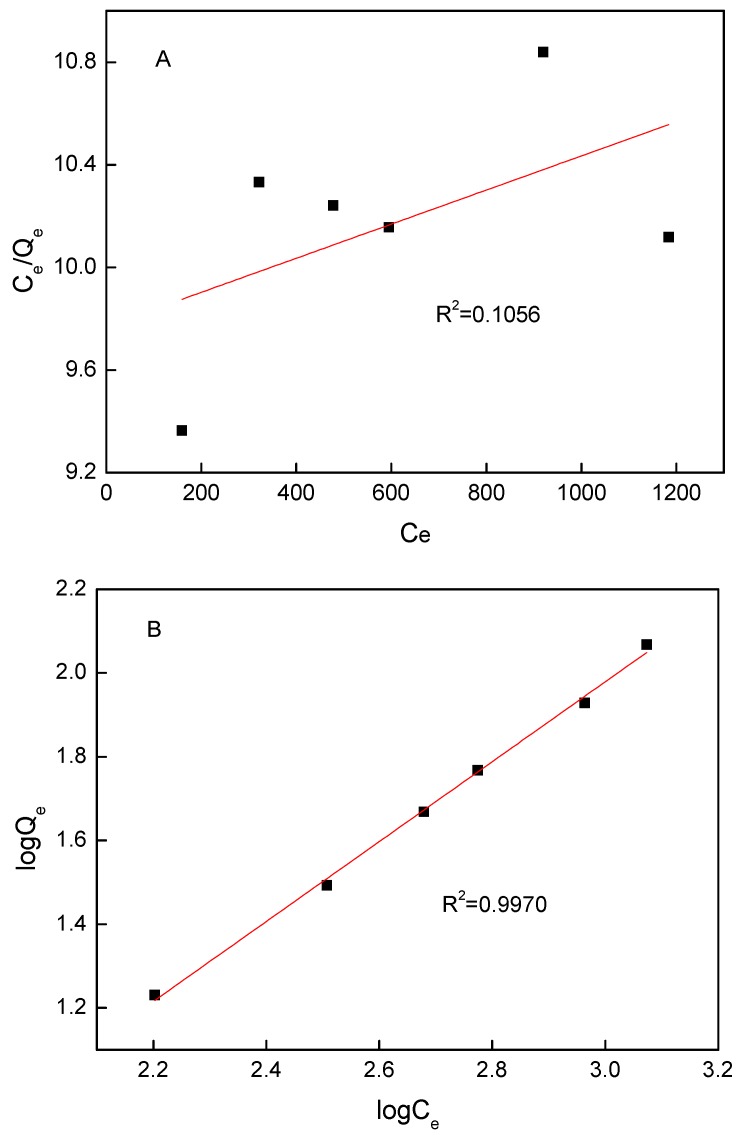
Langmuir (**A**) and Freundlich (**B**) adsorption isotherm for the adsorption of Zn^2+^ onto PIL-3.

The adsorption isotherm of Zn^2+^ on PIL-1 was investigated using typical Langmuir and Freundlich adsorption isotherm models [29,30]. The fitting of the two models is presented in Figure 6, and the calculated parameters are listed in Table 4. It can be noted that the adsorption process was more effectively described by the Freundlich adsorption isotherm model (R^2^ = 0.9970) than the Langmuir adsorption isotherm model (R^2^ = 0.1056). This indicates that the adsorption surface of PIL-1 was heterogeneous. The reason for this could be the increasing porosity of PILs during the thermosynthesis process, which conformed to the Freundlich assumption [31]. In addition, the value of *n* (1.1325) showed that the adsorption of Zn^2+^ onto PIL-3 was favorable. 

## 4. Conclusions

Novel PILMs were constructed and used as the in situ synthesis media for the polymerization of PILs. The designed polymers were proved to be useful adsorbents for the removal of Zn^2+^ from aqueous solution. DLS analysis showed the ionic liquid microemulsion nature of the polymerization media and indicated that the dosage of PEGDA affected the average size of the PILMs. The FTIR spectra confirmed the successful preparation of the designed PILs. Thermal analysis showed that the PILs synthesized within PILMs combined the thermogravimetric characteristics of [AMIM][Cl] and [AMIM][PF_6_], showing good thermostability, and the *T_g_* of PILs were about −30 °C. Within the range of study, PIL-3 showed the best thermogravimetric performance. C_0_, pH, adsorbent dosage and contact time affected the adsorption performance of Zn^2+^ on PILs. In addition, the adsorption process was effectively described by the pseudo-second-order kinetic model and the Freundlich isotherm model.

## Figures and Tables

**Table 1:** The general recipe used in the preparation of poly(ionic liquid)s (PILs ^a^).

Sample Names	m_(AMIMCl)_ /g	m_(AMIMPF6)_ /g	m_(HAPS)_ /g	m_(PEGDA)_ /g	m_(AIBN)_ /g	Temp. /°C
**PILM-1**	3.0611	4.0031	6.0065	0.7221	\	60
**PILM-2**	3.0618	4.0243	5.9999	1.0731	\	60
**PILM-3**	3.0481	4.0031	6.0145	1.7653	\	60
**PILM-4**	3.0814	4.0005	6.0062	2.8480	\	60
**PIL-1**	3.0615	4.0207	6.0125	2.8482	1.1188	80
**PIL-2**	3.0588	4.0515	6.0003	2.8548	1.1196	90
**PIL-3**	3.0492	4.0054	6.0082	2.8395	1.1172	100
**PIL-4**	3.0630	4.0943	6.0168	2.8704	1.1361	110
**PIL-5**	3.0346	4.0721	6.0270	2.8555	1.1231	120

^a^ The uncertainty limits are ±0.1%.

**Table 2:** *E_s_* of PILs in water.

PIL	PIL-1	PIL-2	PIL-3	PIL-4	PIL-5
*E_s_*	21.37	21.01	20.82	21.18	20.85

**Table 3:** Adsorption kinetic parameters of Zn^2+^ on PIL-3.

*Q_e,exp_* mg/g	Pseudo-First Order			Pseudo-Second Order			
	*Q_e,cal_* mg/g	*k*_1_	*R* ^2^	*Q_e,cal_* mg/g	*k*_2_ × 10^4^	*H* mg/(g min)	*R* ^2^
273.03	196.17	0.0120	0.8743	312.5	0.5172	5.0505	0.9969

**Table 4:** Adsorption isotherm parameters of Zn^2+^ on PIL-3.

Langmuir Isotherm			Freundlich Isotherm		
*Q_max_* mg/g	*k_L_* L/mg	*R* ^2^	*K*_f_ L/g	*n*	*R* ^2^
0.1505	0.6801	0.1056	8.9991	1.1325	0.9970
